# Qualitative Synthesis of Young People’s Experiences With Technology-Assisted Cognitive Behavioral Therapy: Systematic Review

**DOI:** 10.2196/13540

**Published:** 2019-11-12

**Authors:** Darragh McCashin, David Coyle, Gary O'Reilly

**Affiliations:** 1 School of Psychology University College Dublin Dublin Ireland; 2 School of Computer Science University College Dublin Dublin Ireland

**Keywords:** cognitive behavioral therapy, systematic review, qualitative research, children, mental health, technology, mHealth, eHealth

## Abstract

**Background:**

Cognitive behavioral therapy (CBT) for young people is increasingly being provided using technology-assisted formats. Although there is increasing evidence regarding the efficacy of such approaches, as illustrated by quantitative systematic reviews, the literature has also highlighted challenges with implementation factors, including high attrition rates and variable user engagement. Qualitative review methods can help to address the factors that impact young peoples’ experience of technology-assisted cognitive behavioral therapy (tech-assisted CBT) and, thus, enable us to better understand such implementation factors. To date, no such qualitative synthesis exists.

**Objective:**

The primary aim of this review was to systematically identify and synthesize the qualitative literature concerning the experiences of young people who have used tech-assisted CBT.

**Methods:**

This systematic review applied Thomas and Harden’s 2008 qualitative thematic synthesis approach. This involved line-by-line coding of the results sections of included studies and an inductive analysis on identified themes, followed by the generation of analytical themes through a process of iteration and interpretation of the descriptive themes. PsycINFO, ACM Digital Library, PubMed, EMBASE, and JMIR Publications databases were searched. The inclusion criteria were (1) studies involving school-aged young people over preschool age (6 years) but under the age of 18 years, (2) use of any form of tech-assisted CBT for any time period, (3) a stated focus of qualitative data to document the experiences of participants, and (4) studies published in English. The exclusion criteria were (1) interventions only provided face-to-face with no technological component, (2) only focused on the performance of the technology rather than participant experience, and (3) numerical data that sought to represent qualitative data.

**Results:**

A total of 14 studies were included in this review. Overall, these studies represented interventions for low mood and anxiety (n=10), trauma or self-harm (n=2), and physical difficulties (n=2). Overall, 5 analytical themes emerged on young people’s experiences with tech-assisted CBT: (1) helpfulness, (2) therapeutic process, (3) transferability, (4) gameplay experience, and (5) limitations. In addition, these analytical themes contained the following subthemes: positive experiences, tech-assisted CBT versus face-to-face CBT, understanding of a CBT model, process of change, skills development, application to everyday life settings, parental involvement, character relatedness, playability, negative experiences, and broad content.

**Conclusions:**

Overall, young people’s experiences with tech-assisted CBT were mostly positive. The use of gaming environments, relatable characters, concrete metaphors, and age-appropriate narratives contributed to these positive experiences. Evidence suggests that technology can help to mediate face-to-face relationships with therapists and help young people to understand the CBT model. Clear barriers also emerged, including over-reliance on reading and writing skills and dissatisfaction with overly generalized content and comparison with commercial technologies.

**Trial Registration:**

International Prospective Register of Systematic Reviews (PROSPERO) CRD42018103388; https://www.crd.york.ac.uk/prospero/display_record.php?ID=CRD42018103388

## Introduction

### Background

Cognitive behavioral therapy (CBT) is used in a range of psychological services for different populations. Given the weight of evidence underpinning CBT [1], it has been recommended as the first response to low levels of anxiety and depression [2]. However, because of resource limitations within mental health services and the notable differences to consider when providing CBT to younger populations [3], CBT has been blended with technology to provide greater accessibility. Digital CBT for depression in young people is now provisionally recommended by the National Institute for Health and Care Excellence as the first line of intervention [4]. Multidisciplinary fields, including psychology, psychiatry, health informatics, and human-computer interaction (HCI), have demonstrated the effectiveness of a range of technology-assisted cognitive behavioral therapy (tech-assisted CBT) interventions for adults and young people across different settings, primarily using quantitative approaches, including formalized trials and systematic reviews [5]. Comparatively, there have been limited systematic qualitative reviews concerning the experiences of young people using tech-assisted CBT. This review commences with a brief clarification on the operationalized definition of tech-assisted CBT, followed by a theoretical summary of CBT. Thereafter, the current gap in the literature is outlined in the context of the limitations of effectiveness research. Finally, this review addresses this gap by applying a qualitative thematic synthesis approach to addressing the experiences of young people using tech-assisted CBT in what is, to our knowledge, the first of its kind to date. This is of importance for future tech-assisted CBT design and evaluations by contextualizing how and why tech-assisted CBT interventions are effective across different settings from the perspective of the young people who have used it.

### Defining Technology-Assisted Cognitive Behavioral Therapy

The introduction of first-generation forms of tech-assisted CBT, commonly referred to as computerized cognitive behavioral therapy (cCBT), was motivated by the need to address the accessibility challenges within mental health services provision. Where demand for such services exceeded the supply, cCBT was primarily envisioned to be a viable method to enhance the reach of evidence-based low-intensity interventions, in addition to being a cost-effective means of doing so [6]. Many of these interventions targeted adults and were mainly internet-based with minimal face-to-face contact [7]. However, within this period, the focus on the potential user experience brought about through providing CBT via technology or on the potentially unintended outcomes and experiences of key stakeholders was less.

This has led to a new wave of different tech-assisted CBT interventions. In addition to acknowledging the economic and accessibility advantages of these interventions, this new wave of tech-assisted CBT has identified the need to continually optimize the design of technologies, learn from user experiences, and measure implementation variables to maximize and sustain positive outcomes [8]. In addition, there is now a wider consideration of the factors that may differentiate tech-assisted CBT from traditional face-to-face CBT, including potentially novel therapeutic barriers and facilitators [9-11]. As a result, there is now a diverse range of interventions that use different technological ingredients to support the CBT process, including mobile phone and tablet apps [12], game-based software [13], interactive websites [14], virtual reality [15], and telecommunications [16]. Increasingly, these new interventions are embedded within a stepped care model [17].

Therefore, tech-assisted CBT can be defined in a variety of ways. Indeed, several descriptors for this intervention are observed throughout the literature, including internet-CBT, cCBT, CBT apps, CBT games, tele-CBT, and virtual reality CBT. For the purposes of this review, tech-assisted CBT is broadly considered as any CBT-based intervention that uses technology to facilitate, support, supplement, or replace traditional face-to-face CBT.

### Theoretical Overview

The theoretical foundation to cognitive behavioral theory posits that there is a complex relationship between one’s thoughts, feelings, and behaviors (TFBs) [18]. Beck [19] suggests that, for those experiencing mental or physical difficulties, automatic surface-level thinking (level 1) can often be negative, thereby perpetuating a cycle that can sustain or intensify the initial difficulty. These problematic thinking-feeling-behavior cycles can be maintained via intermediate cognitive processes (level 2), such as memory, attention, beliefs or attitudes, and interpretation. Underpinning these processes is a schematic structure, referred to as *core beliefs,* that implicitly informs one’s worldview, view of others, and view of self. To identify and address each of these interdependent cognitive levels, CBT provides a structured intervention that necessitates the use of metacognition (thinking about one’s thinking) to recognize problematic patterns adversely impacting the individual. As such, CBT represents a complex exercise requiring significant psychoeducational engagement in both therapeutic and real-world settings.

The complexity of the generic CBT model is further amplified when offered to young people who present different developmental needs. For CBT to be effective with young people, it needs to be appropriately tailored to their developmental stage [20]. By using child-friendly content, tech-assisted CBT interventions have the potential to make core CBT concepts more accessible to young people. Relatedly, as the generic cognitive model is underpinned by a highly structured information-processing paradigm, it is particularly amenable to computer-assisted implementation [21]. Indeed, the integration of age-appropriate content within this process using narrative-based metaphors and accessible characters has been suggested as a developmentally appropriate way to scaffold core CBT concepts [22].

### Rationale for Qualitative Synthesis Within Systematic Reviews

The *gold standard* methodological approach favored by the human and social sciences has long been the randomized controlled trial (RCT) [23]. The procedures stipulated by RCTs allow for the minimization of bias and, thus, the detection of valid and reliable intervention effects. Within behavioral sciences, it is also recognized that high-quality RCTs can have a qualitative component embedded to meaningfully complement the overall dataset [24]. The quantitative method for evaluating the overall effectiveness of an intervention is meta-analysis. This allows for a robust and cumulative appraisal of the effectiveness of an intervention across many studies [25]. Overall, these quantitative approaches significantly contribute to building and (re)evaluating evidence bases and, thus, scientific advancement and eventual impactful policy [26]. However, the qualitative component of outcome studies has often not been included in traditional systematic reviews.

More recently, there has been an advancement of similar high-quality reviewing processes for qualitative data [27]. This has occurred in response to the sometimes restrictive nature of RCTs and what can be missed by not collecting qualitative data. In addition, evidence-based and ethical policy making increasingly necessitates the inclusion of the *voice* of those at whom a policy or intervention is aimed. Indeed, in the area of mental health policy, this is reflected in the embedded role of service user perspectives across research and government institutions [28]. Similarly, in the HCI field, this is observed in participatory or user-centered iterative design approaches [29]. These inclusive methodologies are considered especially relevant for young people, who are often not included within decision-making processes.

Although the apparent objective versus subjective nature of quantitative and qualitative data has long been debated in psychology [30], there has been a notable refinement on how to situate qualitative data within high-quality frameworks of evidence [31]. It is now more conventional to see an evidential framework for an intervention to include a meta-analysis reviewing the current status, followed by an adaptive RCT that is complemented by a qualitative evaluation with participants from within the trial. Such evaluations, therefore, allow for participant experiences, positive and negative feedback, and discursiveness to be aligned with the quantitative outcomes, thereby providing a richer dataset overall.

Despite the proliferation of tech-assisted CBT meta-analyses and RCTs, there have been no qualitative syntheses of the experiences of young people who have used such interventions. Given the vast body of studies demonstrating the feasibility, acceptability, and effectiveness of various tech-assisted CBT interventions [32], this study aimed to synthesize qualitative data from young people who have used tech-assisted CBT for a range of mental and physical issues. Furthermore, this study aimed to collate the overall qualitative themes from these studies to describe the experiences of young people using tech-assisted CBT. Thereafter, the qualitative synthesis can establish analytical themes that can further inform key concepts, understandings, or hypotheses [33]. In doing so, the findings can inform the future design and evaluation of tech-assisted CBT.

### Added Value of Qualitative Synthesis

Systematic reviews that use qualitative synthesis methodology are in keeping with the *moving beyond effectiveness* trajectory within evidence synthesis [34]. Moreover, it is particularly relevant for technology-based interventions by examining the contextual and implementation factors concerning how any intervention is performed and why [8]. Therefore, this study will complement the existing wealth of quantitative studies on tech-assisted CBT by providing an insight into the overall experiences of young people who have used these interventions.

## Methods

A fully accessible protocol for this review was registered on PROSPERO, the international prospective register for systematic reviews [35].

### Search Strategy

A broad search strategy was used across the following databases to capture research bridging psychology, HCI, and related fields: PsycINFO, ACM Digital Library, PubMed, EMBASE, and JMIR Publications (full-text search engine).

The search strategy applied the database-specific methods of using the following search terms: (cognitive OR cognitive therapy OR CBT OR cCBT OR e-therapy OR web-assisted OR tech-assisted OR game-based OR internet) AND (child OR adolescent OR youth OR young people OR young person OR kid OR teen) AND (experience OR perspective OR view OR opinion OR feedback OR interview OR thought OR focus group OR qualitative). A full breakdown of the search terminology is provided in the appendices. In addition, of the selected studies, reference sections were hand-searched for any overlooked literature. The search was conducted in March 2018 and uploaded to Rayyan [36] for storage and screening purposes.

### Inclusion and Exclusion Criteria

Given the wide applicability of tech-assisted CBT, broad inclusion criteria were chosen to facilitate the capture of a wide range of data. As such, the following studies were sought: Studies that are exclusively qualitative by design; studies that have a significant qualitative component alongside quantitative measures; and formal qualitative studies of established or emerging technologies that applied, or sought to apply, tech-assisted CBT.

With respect to the population, the following criteria were applied.

#### Inclusion Criteria

The inclusion criteria were as follows: School-aged young people under the age of 18 years and over the age of preschool (age 6 years); use of any form of tech-assisted CBT-based program for any period (including cCBT, Web-based/assisted CBT, CBT games, CBT mobile apps, blended cCBT, and face-to-face CBT); a stated focus on qualitative data documenting the experiences of the participants that allowed them to express their thoughts, feelings, reflections, both positive and negative feedback, and overall perspectives; and studies published in English.

#### Exclusion Criteria

The exclusion criteria were as follows: CBT is delivered only face to face, with no technological component; only focused on the performance of the technology rather than the participant experience; and numerical data that seek to represent qualitative data.

### Screening and Data Extraction

Owing to the difficulty of locating qualitative data within major databases [37], both the titles and abstracts were screened by DM for potential eligibility. For example, RCTs embed rich qualitative evaluations but may not make this explicit in study abstracts. Following this process, full papers were assessed for eligibility wherein papers that did not utilize qualitative methods could be excluded, and any indecision was recorded via Rayyan. Using the eligibility criteria, 2 independent researchers screened 10% of the overall search and screening by DM. The resulting indecision and disagreement (2%) were discussed and clarified by the review team until consensus was reached.

Thereafter, author DM extracted all data within the *results* or *findings* section that contained direct quotations from the population. It was decided that, as many research papers apply an interpretative voice to qualitative data, this could skew the *voice* of the young person. Therefore, only direct quotes from participants in studies meeting the inclusion criteria were extracted. All quotations were extracted and entered into the qualitative software program NVivo 12 [38].

### Quality Assessment

There is considerable debate surrounding the quality assessment of qualitative research, and even if this can ever be appropriately appraised [39]. There is contention about whether quality assessment should be used to exclude lower-quality studies or to offer a means of assessing the weight of different included studies, given that lower-quality studies can still generate new insights [40]. As there is no consensus regarding methods for excluding studies on the grounds of their interpreted quality [33], all identified studies were included in this review. However, the quality of all included studies was assessed using the 7 quality criteria proposed by Harden et al [41] (see Multimedia Appendix 1), whereby quality is assessed within the context of young people’s views. This refers to the extent to which studies were capable of addressing the review question concerning young people’s overall tech-assisted CBT experiences. All studies were critically appraised on the criteria relating to the theoretical framework, clear statements of study aims, clear context description, clear description of sample and its recruitment, clear description of methods and analysis, validity and reliability, and originality of data.

### Thematic Synthesis

We have selected the thematic synthesis approach laid out by Thomas and Harden [33]. This method involves (1) a line-by-line coding of each papers’ results section, (2) identification of descriptive themes, and (3) generating analytical themes that extend the initial findings. All quotations were coded line by line and the development of descriptive themes occurred by employing an inductive approach using the *Query* and *Explore* functions within *NVivo*. Following the grouping of these descriptive codes, analytical themes were developed by DM alongside the research team using an iterative process of interpretation and reinterpretation of all descriptive themes and supporting quotations. In line with the Thomas and Harden approach, a process of inference throughout these steps generated the analytical themes for the overall experiences of young people and tech-assisted CBT. The resulting analytical themes should be interpreted by the variety and richness of experiences reported rather than the quantification of quotes.

## Results

### Overview

**Figure 1:**
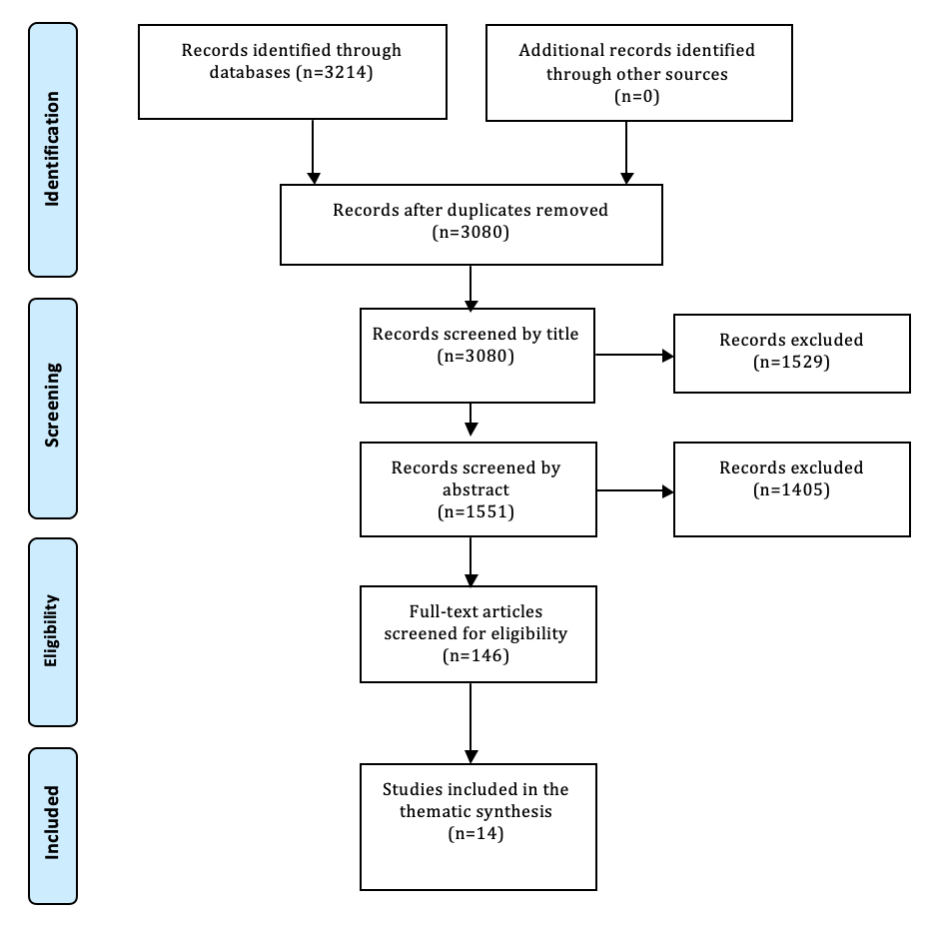
Preferred Reporting Items for Systematic Reviews and Meta-Analyses flowchart.

The search strategy produced 3214 records. Using the Preferred Reporting Items for Systematic Reviews and Meta-Analyses flowchart (Figure 1), 14 studies were deemed eligible for inclusion. These 14 studies comprised data that investigated the experiences of young people with tech-assisted CBT for low mood or anxiety (n=10), trauma or self-harm (n=2), and physical difficulties (n=2). All studies were published between 2013 and 2018. A significant portion of the studies originated from New Zealand (n=6), but there was also broader international coverage with studies from the United States (n=2), the United Kingdom (n=2), the Republic of Ireland (n=1), Sweden (n=1), South Africa (n=1), and Spain (n=1). Of these studies, 9 different types of tech-assisted CBT interventions were investigated within a combined sample of 289 young people. A full breakdown of the characteristics for each study is provided in Multimedia Appendix 2 [42-68] with details of the intervention type, aims and context in which it was applied, participant characteristics, methods, and key themes reported.

Overall, 5 analytical themes describing young people’s experiences of tech-assisted CBT arose, with each of these containing several descriptive themes. To illustrate this synthesis, a table of selected supporting quotations from across the studies is provided (Table 1) in addition to a thematic summary diagram (see Multimedia Appendix 3).

**Table 1:** Supporting quotation for 5 analytical themes and their descriptive subthemes.

Analytical themes		Quotations
**1) The helpfulness of tech-assisted** **cognitive behavioral therapy** **(CBT) for young people**		
	Positive experiences	“It was really good and helpful. Like when I needed to calm down using the technique, it actually helped a lot. Yes, so overall it was pretty good, the breathing in out one.” [54]
	Tech-assisted CBT versus face-to-face CBT	“It’s just cool...it’s a different way...because you know, you go to a counselor and stuff and they have all these different ways of doing things but like, nobody’s ever really thought of a computer game or something. It’s usually like ‘tell me how you’re feeling,’ or ‘write it down’ and stuff, but not ‘play it’.” [49]
**2)** **The therapeutic process within tech-assisted CBT for young people**		
	Understanding CBT model	“I really liked the diary ‘cos...I dunno, ‘cos especially when I’m feeling down I’m like ‘Oh I’m always so sad’ or ‘what is the point’ but actually if I look back to the diary I can see that there were days when I was happy.” [66]
	Process of change	“Oh yeah, I used to get some of those Gnats [gloomy negative automatic thoughts]—but now I am all positive. I am thinking positive, thinking of all the good stuff that has happened to me. I don’t even think about suicide or self-harm now.” [13]
	Skills development	“If I take myself out of the situation and just go sit somewhere else just by myself and focus on breathing, that helps a lot.” [68]
**3)** **The transferability of tech-assisted CBT for young people**		
	Application to everyday life settings	“I have used BlueIce every day. I used the mood checker every day and found it quite easy to use.” [66]
	Parental involvement	“Well, like during the holidays me and my Mum are going to do it together because she is going through a lot of stress as well. We would both sit down and do it and sort it out. I told Mum about the thing [SPARX] and she said that she would like to try it. I am hoping it will actually help her out and take less off her shoulders.” [54]
**4)** **The tech-assisted CBT gameplay experience for young people**		
	Character relatedness	“The thing I kind of liked about it [CCAL] was that he [referring to Charlie] was kind of going through some anxiety too, so you could see it from his point of view.” [58]
	Playability	“I play heaps of games, there is lots of actions, compared with other games SPARX is not great.” [13]
**5)** **The limitations of tech-assisted CBT for young people**		
	Negative experiences	“I might just sort of feel a bit wilful, sort of, somewhat want to stop myself from self-harming but sometimes I just want to self-harm and that’s the end of it really.” [66]
	Broad content	“It was hard because it was catered towards a wider audience and because of that I wasn’t as conﬁdent that it would help my problem.” [68]

### Theme 1: Helpfulness of Technology-Assisted Cognitive Behavioral Therapy for Young People

This theme outlined the varied ways in which young people experienced tech-assisted CBT to be helpful. Young people reported a range of positive experiences in using the technology for mental or physical health difficulties. This analytical theme contains the descriptive subthemes of positive experiences and tech-assisted CBT versus face-to-face CBT.

#### Positive Experiences

There were a large number of positive experiences reported by young people in several studies related to why they found interventions helpful (study numbers 1, 4, 6, 7, 9, and 10). These included a sense of enjoyment and fun during tech-assisted CBT sessions, meaningfulness, feeling motivated, feeling safe, hopefulness, and flexibility.

#### Technology-Assisted Cognitive Behavioral Therapy Versus Face-to-Face Cognitive Behavioral Therapy

It was evident that some young people were engaged with tech-assisted CBT because of their expressed preference for it compared with traditional face-to-face interventions. The ability to use tech-assisted CBT (or adjuncts to tech-assisted CBT, such as apps) encouraged help-seeking behaviors among young people (5 and 12). Moreover, young people felt that technology was easier to engage with than speaking with adults; this was because of the feeling of less stigmatization, the ability to control the pace of the game, and disliking talking (1, 3, 4, 7, and 10).

### Theme 2: Therapeutic Process Within Technology-Assisted Cognitive Behavioral Therapy for Young People

This next theme provided an insight into the ways in which the therapeutic process was observed in young people’s reported experiences. The underlying descriptive themes were understanding the CBT model, process of change, and skills development.

#### Understanding the Cognitive Behavioral Therapy Model

Numerous studies illustrated that young people understood key CBT concepts (1, 6, 7, and 12). Young people spoke about the role of thinking about one’s thinking and recognizing negative thoughts and feelings without judging oneself.

#### Process of Change

Related to the above theme, young people were evidently applying CBT concepts to enact change as well. Several studies highlighted how young people’s experiences of applying positive coping mechanisms and noticing negative automatic thoughts before they manifested as negative behaviors led to positive change in their home, school, or family life (3, 6, 12, and 14). Another study using tech-assisted CBT for trauma indicated that young people were able to utilize tech-assisted CBT technology to confront difficult memories to elicit the therapeutic process of understanding and overcoming them (11).

#### Skills Development

Participants across the studies indicated that many of the skills taught within tech-assisted CBT were directly therapeutic to them. Young people spoke about how focusing on breathing or progressive muscular relaxation exercises were all very helpful and made them feel calmer (6, 8, and 14).

### Theme 3: Transferability of Technology-Assisted Cognitive Behavioral Therapy for Young People

The recognition from young people about the potential transferability of tech-assisted CBT in both their lives and the lives of others was a further analytical theme in the data. Young people communicated a variety of ways in which tech-assisted CBT could be transferred, as explored in 2 descriptive themes: application to everyday life settings and parental involvement.

#### Application to Everyday Life Settings

Young people were able to reflect on their own experiences with tech-assisted CBT. They also believed tech-assisted CBT could be applied in everyday life settings (1, 3, 6, and 12). Nonetheless, young people were mindful of some caveats to this. Young people mentioned that having components of tech-assisted CBT to share with others would be beneficial, but that some aspects of the content they used within tech-assisted CBT may not be applicable in some settings, such as school or family settings (unless privacy and discreteness were ensured). Indeed, young people acknowledged that tech-assisted CBT is applicable for many young people experiencing mental or physical health difficulties, but not all young people.

#### Parental Involvement

A small number of young people noticed the potential concurrent role that tech-assisted CBT could play for their parents, either as a support for their own progress or as an intervention specifically for parents (5, 10, and 12).

### Theme 4: Technology-Assisted Cognitive Behavioral Therapy Gameplay Experience for Young People

The fourth theme in the synthesis is related to the features implicit in the gameplay or user experience of the respective technologies in this review. The 2 underpinning descriptive themes were character relatedness and playability.

#### Character Relatedness

Young people were mostly appreciative of the characters embedded within many of the tech-assisted CBT interventions (1, 3, 4, 5, 6, and 9). Many felt that the characters were relatable and that they allowed the communication of CBT content to, therefore, be easier. Notably, in studies concerning minority groups (1 and 3-6), this was especially relevant to participants who were pleased with the inclusion of Maori identities in the game. In addition, this descriptive theme extended to the personalization of young people’s own role within the game. The autonomy afforded to young people in selecting their interactions with characters or personalizing avatars was considered an important gameplay feature.

#### Playability

Though only a minor theme, there were some mixed experiences from young people in relation to the actual playability of some tech-assisted CBT interventions (1, 3, 4, 6, 7, and 12). Some young people expressed the view that tech-assisted CBT was lacking in its gaming experience, especially when compared with commercial games. However, other young people felt that playing tech-assisted CBT games was easy, familiar, and intuitive.

### Theme 5: Limitations of Technology-Assisted Cognitive Behavioral Therapy for Young People

This final theme gave an insight into the limitations of tech-assisted CBT that young people experienced. Despite many positive findings in the preceding themes, young people were also aware of the drawbacks of tech-assisted CBT. Within this, 2 descriptive themes were recorded: negative experiences and broad content.

#### Negative Experiences

There were a number of studies with a notably small number of young people that outlined some negative experiences with tech-assisted CBT (1, 4, 7, 8, and 12). Some young people stated that there remains a stigma associated with using tech-assisted CBT and that it is challenging to discuss psychological or physical problems with an adult, irrespective of the presence of tech-assisted CBT. Other negative experiences that were mentioned included feeling worse on occasions, not experiencing change, feeling frustrated when returning to face-to-face therapy, and feeling distracted.

#### Broad Content

Finally, a negligible descriptive theme arose in relation to the nature of the content in tech-assisted CBT (5, 7, and 14). Some young people identified the broad content of the psychoeducational materials to be problematic and sometimes confusing. Similarly, too much reading and writing content was also seen as a limitation of tech-assisted CBT.

## Discussion

### Why Do These Themes Matter?

The aim of this study was to synthesize the qualitative literature regarding the experiences of young people who have used tech-assisted CBT for a range of mental or physical difficulties. Using a qualitative synthesis approach, 5 analytical themes and 11 descriptive themes emerged from the included studies containing a diverse sample of young people. Overall, these themes can be understood to be conceptually interrelated and are of interest for several reasons.

Taken together, themes 1 through 4 can be interpreted to broadly support the theoretical foundations of CBT, as suggested by the supporting quotation (Table 1). Young people’s reported experiences of using exercises connecting TFBs were associated with themes of helpfulness and the therapeutic process. Many young people were able to think about the connections between their surface-level TFBs (“Oh yeah, I used to get some of those gnats”) and engage with deeper cognitive processes including memory and attention (“...actually if I look back to the diary I can see that there were days when I was happy”), alongside some suggestions of developing more beneficial core beliefs (“but now I am all positive”). Furthermore, as effective CBT requires appropriate tailoring for young people [20], this review provides evidence that tech-assisted CBT is one effectual approach of doing so. Many of the interventions that were reviewed had applied unique ways of using technology to connect with young people, including the use of personalized avatars, child-friendly narratives, and computer game–based designs, with some young people specifying these factors as relatable (“he [referring to character] was kind of going through some anxiety too”).

The first theme—the helpfulness of tech-assisted CBT for young people—corroborated the dominant trend evident in the quantitative literature, that is, young people who completed tech-assisted CBT interventions were significantly more likely to report positive outcomes versus control groups [5]. This review has provided the context to these positive trends by synthesizing what young people are saying about tech-assisted CBT, thereby offering an insight into why tech-assisted CBT is proving effective. It should be noted that young people reported the following reasons as to why tech-assisted CBT was helpful for them: they described a range of positive experiences within tech-assisted CBT and appeared to favor it over traditional face-to-face interventions. Moreover, the second analytic theme demonstrated that a range of young people in different settings experienced a therapeutic process that facilitated positive change. This is in keeping with the intended theory-to-practice model applied by core CBT approaches. Indeed, that young people highlighted the unique characteristics of tech-assisted CBT over face-to-face interventions underscored the value of offering tech-assisted CBT to appeal to young people. This is noteworthy because of the many concerns about the increasing role of technology in CBT interventions and its effect on the attitudes, acceptability, and uptake from different stakeholders over time [69,70]. This synthesis broadly validates the incorporation of technology into mental health interventions from the perspective of young people’s reported experiences.

A further theme that was of interest was young people’s perceived transferability of tech-assisted CBT in not only their lives but also the lives of others. Related to the therapeutic process discussed above, young people were able to recognize that their experiences in completing tech-assisted CBT were directly applicable to their different roles in school and with family and friends, and they were able to evidence these with examples. By extension, although it was a minor theme, it was also felt that parental involvement could be explored further within tech-assisted CBT interventions. This is of relevance given the potential relationship between parental anxiety and child anxiety [71], but it also suggests that there could be future adaptations of tech-assisted CBT that could further engage parents or other stakeholders. It should be noted that young people provided both supportive and unsupportive feedback regarding the involvement of parents. Similarly, young people provided several caveats to the transferability of tech-assisted CBT (eg, only some of their tech-assisted CBT content would be appropriate, and there were concerns about privacy). These considerations may be suggestive of potential subgroups of young people that might benefit from different adaptations of tech-assisted CBT that could support their individualized needs and preferences to maximize the transferability of tech-assisted CBT to key areas in their lives. As concerns remain about the long-term effectiveness of tech-assisted CBT beyond their evaluative timeframes [72], these suggestions could be especially important given that tech-assisted CBT can only maintain its effectiveness if core CBT principles are transferred to everyday life. For example, relapse-prevention strategies within tech-assisted CBT necessitate the effective transfer of CBT psychoeducation to the respective areas of young people’s lives. Therefore, any strategies that can support this transfer should be explored. Nonetheless, it was apparent that young people in this review were widely transferring lessons from their tech-assisted CBT experiences into different areas of their lives.

In the context of traditional mental health interventions, tech-assisted CBT is unique given its technological mode of delivery, often guided in person by appropriately trained mental health professionals. The fourth theme—the tech-assisted CBT gameplay experience—was particularly insightful regarding the benefits of using gaming technology to support interventions. There was widespread appeal of tech-assisted CBT characters for young people, which was found to be a key factor in fostering a sense of relatedness and autonomy. This was particularly noticeable in the smart, positive, active, realistic, X-factor thoughts (SPARX) adaptations for different minority groups, where character relatedness was a recurring subtheme discussed within young people’s positive experiences. This is an important dynamic because of the influential role of social learning for young people [73] and reinforces the need for continued development of personalization features for tech-assisted CBT characters that span youth cultures and identities. As a result, the experiences young people described were demonstrative of enhanced engagement with CBT content, partly because of the gameplay mechanism of interaction. However, this must be considered alongside some mixed feedback regarding the playability features of tech-assisted CBT where young people observed a contrast with commercial games. This is a worthwhile comparison for tech-assisted CBT facilitators as these contrasts could develop into intervention resistance or disengagement. This also suggests that managing young people’s expectations for tech-assisted CBT is important in optimizing their subsequent engagement; however, no standardized approaches for doing so were evident in this review.

The final theme emphasized some of the limitations of tech-assisted CBT from the perspective of young people. Although this was also a minor theme, young people commented on the sometimes broad content of tech-assisted CBT in addition to a small number of negative experiences—a finding echoed in the research on adult experiences [9]. The precise reasons as to why these themes arose were unclear, but it is advisable that stakeholders factor in the persistent role of stigmatization and intervention nonresponse for a minority of young people.

### Limitations

Compared with the quantitative literature, there was a significant lack of qualitative studies on this topic. Consequently, only 14 studies were included in this review. Although this proved sufficient to extract a threshold of data, the combined dataset was still relatively small in comparison with meta-analyses. In addition, most of the studies involved young people who had consented to research and who had completed their intervention. This means that the data did not include young people who may have dropped out or were incapable of research participation because of psychological or physical difficulties. Although only 1 analytical theme reflected negative experiences, it is possible that these attrition challenges reduced the ability of this review to identify the negative aspects of tech-assisted CBT or new findings related to tech-assisted CBT experiences for young people. The reasons for such attrition or nonparticipation are especially important in the critical appraisal of the uptake of tech-assisted CBT but were beyond the scope of this review. Had there been qualitative studies analyzing the experiences of those who dropped out or disliked tech-assisted CBT, a richer thematic structure regarding negative experiences or unintended consequences would have emerged.

It should also be noted that a large portion of the included studies come from 1 intervention (SPARX) that has a significant evidence base demonstrating its effectiveness. As such, the overall synthesis may be disproportionately attributable to the characteristics of this intervention. Moreover, some of these studies pertained to the design or prototyping of the intervention [43,49,51], and therefore, they may be liable to desirability biases that do not reflect the experiences of real-world adherence. This disproportionate coverage also reiterates the lack of qualitative studies for a variety of tech-assisted CBT interventions across the extant literature.

A further limitation is that only direct quotes from young people were included in the analysis and not the full results sections of papers. Although this decision was made to ensure that only the voice of young people was analyzed, it may have limited the potential insights and reliability of the overall synthesis.

In addition, as this review purposively used a broad definition of tech-assisted CBT, it is not known if the reported themes would emerge with specific variants of CBT—for example, are game-based designs of tech-assisted CBT experienced significantly differently to Web-based or app-based versions?

### Future Research and Recommendations

Future research would benefit from further well-designed qualitative studies that focus exclusively on the experiences of young people. Specifically, studies should endeavor to also include the experiences of young people who find tech-assisted CBT to be unhelpful or those who drop out. This would be particularly useful in ascertaining what intervention adaptations could be made or *built in* to the technology to mitigate against attrition risks. The continued debate about the merits of qualitative research could be addressed by the implementation of enhanced quality assessment for evidence syntheses. In addition, the use of standardized questions when evaluating tech-assisted CBT could enhance the comparability of reviews.

As the tech-assisted CBT experience could be both positively or negatively affected by the absence of common factors that typify face-to-face CBT, this review has provided an insight into, and support for, the suggestion that computerized therapies, such as tech-assisted CBT, have their own unique common factors [9]. In particular, the subthemes discussed in this review illustrated novel factors brought about because of the technological component of CBT (namely, *tech-assisted CBT vs face-to-face CBT*, *character relatedness*, and *playability*). Therefore, in addition to appropriately applying the underlying CBT theory, future research and design can improve positive outcomes for young people by focusing on these novel factors. In practice, this means that designers of tech-assisted CBT should consider how to best enhance the personalization process for young people, the sophistication of child-friendly and psychologically appropriate narratives or avatars, and maximizing pathways for young people to transfer their learning to everyday life. For tech-assisted CBT practitioners, future research needs to further develop our qualitative understanding of these novel factors and how they relate to the therapeutic process, successful implementation of tech-assisted CBT, and the early identification of potential intervention nonresponders.

## Appendix

Multimedia Appendix 1 Critical appraisal of included studies using criteria proposed by Harden et al.

Multimedia Appendix 2 Characteristics of included studies.

Multimedia Appendix 3 Thematic summary diagram.

## Abbreviations

CBT: cognitive behavioral therapy
cCBT: computerized cognitive behavioral therapy
HCI: human-computer interaction
RCT: randomized controlled trial
SPARX: smart, positive, active, realistic, X-factor thoughts
TEAM: Technology Enabled Mental Health for Young People
tech-assisted CBT: technology-assisted cognitive behavioral therapy
TFBs: thoughts, feelings, and behaviors

## References

[1] Herbert JD, Forman EM (2011). Acceptance and Mindfulness in Cognitive Behavior Therapy: Understanding and Applying the New Therapies.

[2] National Collaborating Centre for Mental Health (2005). Depression in Children and Young People: Identification and Management in Primary, Community and Secondary Care.

[3] Grave J, Blissett J (2004). Is cognitive behavior therapy developmentally appropriate for young children? A critical review of the evidence. Clin Psychol Rev.

[4] Wise J (2019). Depression in children: offer digital CBT as first line treatment, says NICE. Br Med J.

[5] Pennant ME, Loucas CE, Whittington C, Creswell C, Fonagy P, Fuggle P, Kelvin R, Naqvi S, Stockton S, Kendall T, Expert Advisory Group (2015). Computerised therapies for anxiety and depression in children and young people: a systematic review and meta-analysis. Behav Res Ther.

[6] Gerhards SA, de Graaf LE, Jacobs LE, Severens JL, Huibers MJ, Arntz A, Riper H, Widdershoven G, Metsemakers JF, Evers SM (2010). Economic evaluation of online computerised cognitive-behavioural therapy without support for depression in primary care: randomised trial. Br J Psychiatry.

[7] Cavanagh K, Shapiro DA (2004). Computer treatment for common mental health problems. J Clin Psychol.

[8] Mohr DC, Riper H, Schueller SM (2018). A solution-focused research approach to achieve an implementable revolution in digital mental health. JAMA Psychiatry.

[9] Knowles SE, Toms G, Sanders C, Bee P, Lovell K, Rennick-Egglestone S, Coyle D, Kennedy CM, Littlewood E, Kessler D, Gilbody S, Bower P (2014). Qualitative meta-synthesis of user experience of computerised therapy for depression and anxiety. PLoS One.

[10] Henson P, Wisniewski H, Hollis C, Keshavan M, Torous J (2019). Digital mental health apps and the therapeutic alliance: initial review. BJPsych Open.

[11] Sucala M, Schnur JB, Constantino MJ, Miller SJ, Brackman EH, Montgomery GH (2012). The therapeutic relationship in e-therapy for mental health: a systematic review. J Med Internet Res.

[12] Torous J, Powell AC (2015). Current research and trends in the use of smartphone applications for mood disorders. Internet Interv.

[13] Fleming TM, Bavin L, Stasiak K, Hermansson-Webb E, Merry SN, Cheek C, Lucassen M, Lau HM, Pollmuller B, Hetrick S (2016). Serious games and gamification for mental health: current status and promising directions. Front Psychiatry.

[14] Spek V, Cuijpers P, Nyklícek I, Riper H, Keyzer J, Pop V (2007). Internet-based cognitive behaviour therapy for symptoms of depression and anxiety: a meta-analysis. Psychol Med.

[15] Scozzari S, Gamberini L, Brahnam S, Jain LC (2011). Virtual reality as a tool for cognitive behavioral therapy: a review. Advanced Computational Intelligence Paradigms in Healthcare 6. Virtual Reality in Psychotherapy, Rehabilitation, and Assessment.

[16] Lovell K, Cox D, Haddock G, Jones C, Raines D, Garvey R, Roberts C, Hadley S (2006). Telephone administered cognitive behaviour therapy for treatment of obsessive compulsive disorder: randomised controlled non-inferiority trial. Br Med J.

[17] Green KE, Iverson KM (2009). Computerized cognitive-behavioral therapy in a stepped care model of treatment. ‎Prof Psychol: Res Pract.

[18] Beck AT (1963). Thinking and depression. I. Idiosyncratic content and cognitive distortions. Arch Gen Psychiatry.

[19] Beck AT, Dozois DJ (2011). Cognitive therapy: current status and future directions. Annu Rev Med.

[20] Carr A (2008). What Works with Children, Adolescents, and Adults?: A Review of Research on the Effectiveness of Psychotherapy.

[21] Coyle D, McGlade N, Doherty G, O'Reilly G (2011). Exploratory Evaluations of a Computer Game Supporting Cognitive Behavioural Therapy for Adolescents. Proceedings of the SIGCHI Conference on Human Factors in Computing Systems.

[22] O'Reilly G, Pesky G, Harnish RJ, Bridges KR, Sattler DN, Signorella ML, Munson M (2018). Pesky gNATs! Using computer games and smartphone apps to teach complex cognitive behavioural therapy and mindfulness concepts to children with mental health difficulties. The Use of Technology in Teaching and Learning.

[23] Torgerson DJ, Torgerson CJ (2008). Designing Randomised Trials in Health, Education and the Social Sciences: An Introduction.

[24] O'Cathain A, Thomas KJ, Drabble SJ, Rudolph A, Hewison J (2013). What can qualitative research do for randomised controlled trials? A systematic mapping review. BMJ Open.

[25] Borenstein M, Hedges LV, Higgins JP, Rothstein HR (2011). Introduction to Meta-Analysis.

[26] Cartwright N (2012). Evidence-Based Policy: A Practical Guide to Doing It Better.

[27] Noyes J, Booth A, Flemming K, Garside R, Harden A, Lewin S, Pantoja T, Hannes K, Cargo M, Thomas J (2018). Cochrane qualitative and implementation methods group guidance series-paper 3: methods for assessing methodological limitations, data extraction and synthesis, and confidence in synthesized qualitative findings. J Clin Epidemiol.

[28] Thornicroft G, Tansella M (2005). Growing recognition of the importance of service user involvement in mental health service planning and evaluation. Epidemiol Psichiatr Soc.

[29] Terp M, Laursen BS, Jørgensen R, Mainz J, Bjørnes CD (2016). A room for design: through participatory design young adults with schizophrenia become strong collaborators. Int J Ment Health Nurs.

[30] Cromby J (2011). Feeling the way: qualitative clinical research and the affective turn. Qual Res Psychol.

[31] Hannes K, Macaitis K (2012). A move to more systematic and transparent approaches in qualitative evidence synthesis: update on a review of published papers. Qual Res.

[32] Ebert DD, Zarski AC, Christensen H, Stikkelbroek Y, Cuijpers P, Berking M, Riper H (2015). Internet and computer-based cognitive behavioral therapy for anxiety and depression in youth: a meta-analysis of randomized controlled outcome trials. PLoS One.

[33] Thomas J, Harden A (2008). Methods for the thematic synthesis of qualitative research in systematic reviews. BMC Med Res Methodol.

[34] Popay J (2006). Moving Beyond Effectiveness In Evidence Synthesis: Methodological Issues in the Synthesis of Diverse Sources of Evidence.

[35] McCashin D, O'Reilly G, Coyle D (2018). PROSPERO - University of York.

[36] Ouzzani M, Hammady H, Fedorowicz Z, Elmagarmid A (2016). Rayyan-a web and mobile app for systematic reviews. Syst Rev.

[37] Finfgeld-Connett D, Johnson ED (2013). Literature search strategies for conducting knowledge-building and theory-generating qualitative systematic reviews. J Adv Nurs.

[38] Richards L (1999). Using Nvivo In Qualitative Research.

[39] Thomas J, Harden A, Oakley A, Oliver S, Sutcliffe K, Rees R, Brunton G, Kavanagh J (2004). Integrating qualitative research with trials in systematic reviews. Br Med J.

[40] Shuster JJ, Higgins JP, Green S (2011). Cochrane collaborations. Cochrane Handbook for Systematic Reviews of Interventions Version 5.1.0.

[41] Harden A, Garcia J, Oliver S, Rees R, Shepherd J, Brunton G, Oakley A (2004). Applying systematic review methods to studies of people's views: an example from public health research. J Epidemiol Community Health.

[42] Lucassen MF, Hatcher A, Fleming TM, Stasiak K, Shepherd MJ, Merry SN (2015). A qualitative study of sexual minority young people's experiences of computerised therapy for depression. Australas Psychiatry.

[43] Lucassen MF, Hatcher S, Stasiak K, Fleming T, Shepherd M, Merry SN (2014). The views of lesbian, gay and bisexual youth regarding computerised self-help for depression: an exploratory study. Advances in Mental Health.

[44] Lucassen MF, Merry SN, Hatcher S, Frampton CM (2015). Rainbow SPARX: a novel approach to addressing depression in sexual minority youth. Cogn Behav Pract.

[45] Merry SN, Stasiak K, Shepherd M, Frampton C, Fleming T, Lucassen MF (2012). The effectiveness of SPARX, a computerised self help intervention for adolescents seeking help for depression: randomised controlled non-inferiority trial. Br Med J.

[46] Thomas DR (2016). A general inductive approach for analyzing qualitative evaluation data. Am J Eval.

[47] Poznanski EO, Grossman JA, Buchsbaum Y, Banegas M, Freeman L, Gibbons R (1984). Preliminary studies of the reliability and validity of the children's depression rating scale. J Am Acad Child Psychiatry.

[48] Fleming T, Lucassen M, Stasiak K, Shepherd M, Merry S (2015). The impact and utility of computerised therapy for educationally alienated teenagers: the views of adolescents who participated in an alternative education-based trial. Clin Psychol.

[49] Cheek C, Bridgman H, Fleming T, Cummings E, Ellis L, Lucassen MF, Shepherd M, Skinner T (2014). Views of young people in rural Australia on SPARX, a fantasy world developed for New Zealand youth with depression. JMIR Serious Games.

[50] Boyatzis RE (1998). Transforming Qualitative Information: Thematic Analysis and Code Development.

[51] Shepherd M, Fleming T, Lucassen M, Stasiak K, Lambie I, Merry SN (2015). The design and relevance of a computerized gamified depression therapy program for indigenous Māori adolescents. JMIR Serious Games.

[52] Gilgun JF (2005). Qualitative research and family psychology. J Fam Psychol.

[53] Braun V, Clarke V (2006). Using thematic analysis in psychology. Qual Res Psychol.

[54] Shepherd M, Merry S, Lambie I, Thompson A (2018). Indigenous adolescents' perception of an eMental health program (SPARX): exploratory qualitative assessment. JMIR Serious Games.

[55] Tunney C, Cooney P, Coyle D, O'Reilly G (2017). Comparing young people's experience of technology-delivered v face-to-face mindfulness and relaxation: two-armed qualitative focus group study. Br J Psychiatry.

[56] O'Reilly G, Coyle D (2015a). http://www.io.nihr.ac.uk/wp-content/uploads/migrated/2626.6251b4c8.PeskygNATsCBTgameFINAL.pdf.

[57] Chapman R, Loades M, O'Reilly G, Coyle D, Patterson M, Salkovskis P (2016). ‘Pesky gNATs’: investigating the feasibility of a novel computerized CBT intervention for adolescents with anxiety and/or depression in a tier 3 CAMHS setting. Cogn Behav Ther.

[58] Salloum A, Crawford EA, Lewin AB, Storch EA (2015). Consumers' and providers' perceptions of utilizing a computer-assisted cognitive behavioral therapy for childhood anxiety. Behav Cogn Psychother.

[59] Khanna MS, Kendall PC (2008). Computer-assisted CBT for child anxiety: the coping Cat CD-ROM. Cogn Behav Pract.

[60] Padgett DK (1998). Qualitative Methods in Social Work Research: Challenges and Rewards.

[61] Lenhard F, Vigerland S, Engberg H, Hallberg A, Thermaenius H, Serlachius E (2016). 'On my Own, but not Alone' - adolescents' experiences of internet-delivered cognitive behavior therapy for obsessive-compulsive disorder. PLoS One.

[62] Scahill L, Riddle MA, McSwiggin-Hardin M, Ort SI, King RA, Goodman WK, Cicchetti D, Leckman JF (1997). Children's Yale-Brown obsessive compulsive scale: reliability and validity. J Am Acad Child Adolesc Psychiatry.

[63] Seidman I (1991). Interviewing as Qualitative Research: A Guide for Researchers in Education and the Social Sciences. Third Edition.

[64] Schilling J (2006). On the pragmatics of qualitative assessment. Eur J Psychol Assess.

[65] Kruger D, Swanepoel M (2017). Gluing the pieces together: female adolescents’ construction of meaning through digital metaphoric imagery in trauma therapy. Art Psychother.

[66] Grist R, Porter J, Stallard P (2018). Acceptability, use, and safety of a mobile phone app (BlueIce) for young people who self-harm: qualitative study of service users' experience. JMIR Ment Health.

[67] Nieto R, Hernández E, Boixadós M, Huguet A, Beneitez I, McGrath P (2015). Testing the feasibility of DARWeb: an online intervention for children with functional abdominal pain and their parents. Clin J Pain.

[68] Law EF, Beals-Erickson SE, Fisher E, Lang EA, Palermo TM (2017). Components of effective cognitive-behavioral therapy for pediatric headache: a mixed methods approach. Clin Pract Pediatr Psychol.

[69] Kaltenthaler E, Sutcliffe P, Parry G, Beverley C, Rees A, Ferriter M (2008). The acceptability to patients of computerized cognitive behaviour therapy for depression: a systematic review. Psychol Med.

[70] Waller R, Gilbody S (2009). Barriers to the uptake of computerized cognitive behavioural therapy: a systematic review of the quantitative and qualitative evidence. Psychol Med.

[71] McLeod BD, Wood JJ, Weisz JR (2007). Examining the association between parenting and childhood anxiety: a meta-analysis. Clin Psychol Rev.

[72] de Graaf LE, Gerhards SA, Arntz A, Riper H, Metsemakers JF, Evers SM, Severens JL, Widdershoven G, Huibers MJ (2011). One-year follow-up results of unsupported online computerized cognitive behavioural therapy for depression in primary care: a randomized trial. J Behav Ther Exp Psychiatry.

[73] Bandura A (1969). Social learning of moral judgments. J Pers Soc Psychol.

