# Supplementary material for: Qualitative Synthesis of Young People’s Experiences With Technology-Assisted Cognitive Behavioral Therapy: Systematic Review
Source: J Med Internet Res. 2019 Nov 12;21(11):e13540. doi: 10.2196/13540 (PMC6880234; doi:10.2196/13540)
Supplement: Multimedia Appendix 1 [file jmir_v21i11e13540_app1.pdf]

Study	Explicit theoretical framework and/or literature review	Aims and objectives clearly stated	A clear description of the context	A clear description of the sample and how it was recruited	A clear description of the methods used to collect and analyse the data	Attempts made to establish the validity of data analysis	Inclusion of sufficient original data to mediate between evidence and interpretation
Lucassen et al. (2015)	✓	✓	✓		✓	✓	✓
Lucassen et al. (2013)	✓	✓	✓		✓	✓	✓
Fleming et al. (2016)	✓	✓	✓	✓	✓	✓	✓
Cheek et al. (2014)	✓	✓	✓		✓	✓	✓
Shepherd et al. (2015)	✓	✓	✓		✓	✓	✓
Shepherd et al. (2018)	✓	✓	✓	✓	✓	✓	✓
Tunney et al. (2017)	✓	✓	✓		✓	✓	✓
Chapman et al. (2016)	✓	✓		✓	✓		✓
Salloum et al. (2015)	✓	✓	✓	✓	✓	✓	
Lenhard et al. (2016)	✓	✓	✓	✓	✓	✓	✓
Kruger & Swanepoel (2017)	✓			✓	✓	✓	

Grist et al. (2018)	✓	✓		✓	✓	✓	✓
Nieto et al. (2018)	✓	✓	✓	✓	✓	✓	
Law et al. (2017)	✓	✓	✓	✓	✓	✓	✓
