# Supplementary material for: Qualitative Synthesis of Young People’s Experiences With Technology-Assisted Cognitive Behavioral Therapy: Systematic Review
Source: J Med Internet Res. 2019 Nov 12;21(11):e13540. doi: 10.2196/13540 (PMC6880234; doi:10.2196/13540)
Supplement: Multimedia Appendix 2 [file jmir_v21i11e13540_app2.pdf]

| Study (country) | Intervention | Aims and context | Sample characteristics (young people data only) | Method | Key themes |
|---|---|---|---|---|---|
| **Technology-assisted cognitive behavioral therapy (CBT) for low mood and anxiety** | | | | | |
| 1. Lucassen et al [42] (New Zealand) | *Rainbow* smart, positive, active, realistic, X-factor thoughts (*SPARX*): This is an adapted version of the original *SPARX* [43-45]. It consists of 7 self-help computerized cognitive behavioral therapy (cCBT) modules using a fantasy world format wherein the user's avatar faces challenges to reduce negative thoughts. | To describe the experiences of lesbian, gay, bisexual (LGB) or sexual minority youth who have used computerized therapy for depression. It sought to determine what participants liked and disliked; how intervention might benefit others; thoughts on content and weekly challenges; and whether or not intervention helped depression. | N=25 (male, n=13; female, n=12: with 2 participants identifying as transgender). Sexual minority adolescents aged 13-19 years were recruited from an open trial with depressive symptoms (using Child Depression Rating Scale-Revised (CDRS-R) raw score ≥30 at baseline) [47], living in Auckland, New Zealand. | Semi-structured interviews, analyzed using the general inductive approach (GIA), a form of qualitative content analysis [46] | 5 main themes: (1) *appealing aspects;* (2) *applying it to real life;* (3) *things to improve;* (4) *aspects that did not appeal; and* (5) *other themes.* Participants suggested more sexuality-specific content be included in the program. Most participants felt the intervention helped them feel better or less depressed (n=17). |

| | | | | | |
|---|---|---|---|---|---|
| 2. Lucassen et al [43] (New Zealand) | *SPARX*: the 2013 version where pretesting of the design, characters, and scenarios of SPARX prototypes were iteratively used to later inform Rainbow SPARX. | To explore the challenges for LGB youth in New Zealand, and to assess how these challenges can be addressed via cCBT | LGB youth recruited from Auckland-based LGB organizations (n=9). Gender identity: *male* (n=2), *female* (n=4), *lesbian* (n=1), m*ale/gender queer* (n=1), gay male (n=1). | 3 focus groups, analyzed using the GIA [46] | 4 main themes regarding LGB challenges: (1) *ways participants can tell that they are "different";* (2) *environments and their impact*; (3) *ways participants cope*; and (4) *depression and sexuality.* 3 themes emerged in relation to *SPARX*: (1) positive comments about the *look and feel* of SPARX; (2) positive comments about the ability for participants to be gender nonconformists; and (3) positive comments about the broader concept of cCBT. |
| 3. Fleming et al [48] (New Zealand) | *SPARX*—cCBT program containing 7 levels of CBT skills taught via direct instruction, a review with a *guide*, and via play-based learning in a fantasy 3-dimensional (3D) game environment | To explore the views of adolescents attending alternative education within the *SPARX* RCT | N=39 (male, n=24; female, n=15), age 13-16 years (mean 14.5, SD 0.76 years). N=30 had symptoms of depression (using CDRS-R raw score ≥30 at baseline) [47], N=9 without. | Semi-structured interviews analyzed using GIA—form of qualitative content analysis [46] | 2 main themes: Personal experience of *SPARX:* overall sample found that it was *fun and helpful*, and *entertaining.* In total, 85% participants described it as helpful ("I used to get some of those Gnats—but now I am all positive"). *SPARX* for others: majority felt that they would recommend it for others, noting it "was freeing" relative to counselling, and encouraged help seeking ("it smoothes the way"). Participants felt it should be offered to all young people, not just help-seeking people. |

| | | | | | |
|---|---|---|---|---|---|
| 4. Cheek et al [49] (New Zealand) | *SPARX*—cCBT program, containing 7 levels of CBT skills taught via direction instruction, a review with a *guide*, and via play-based learning in a fantasy 3D game environment | To explore the acceptability of *SPARX* by rural Australian youth, including their preferred means of access | A total of 16 participants were recruited from community-based services offering early intervention and preventative measures to improve well-being. Participants (75% male) were aged between 13 and 18 years. Reported ethnicity was Aboriginal (n=4), Australian (n=5), and unreported (n=7); 13 participants identified as a *computer gamer.* | Focus groups and semi-structured interviews were conducted. A hybrid approach in developing data-driven code was used for the thematic analysis [50]. | The thematic categories reported were: personalization, engagement, and stigma. |
| 5. Shepherd et al [51] (New Zealand) | *SPARX* prefinalized beta testing stage version, containing content as detailed above | To conduct qualitative research into indigenous New Zealand youth regarding the (then) prototype of *SPARX* | A total of 19 participants were recruited through word of mouth in indigenous (Maori) youth communities (including *taitamariki* and Whanau [families]) aged between 16 and 18 | Focus groups, analyzed using general inductive analysis [52] and thematic analysis [53]. | 4 categorized themes were identified: (1) computerized therapy has good face validity and is seen as potentially effective and appealing; (2) cultural relevance was viewed as being important for the engagement of Maori young people with *SPARX*; (3) Whanau are important for young people's well-being; and (4) ideas for improvement of *SPARX* for |

| | | | | | |
|---|---|---|---|---|---|
| | | | years. | | Maori. |
| 6. Shepherd et al [54] (New Zealand) | *SPARX*—cCBT program containing 7 levels of CBT skills taught via direction instruction, a review with a *guide*, and via play-based learning in a fantasy 3D game environment. | To conduct exploratory qualitative research of Maori adolescents' views about a cCBT program (*SPARX*). | Six young people (1 male) aged 14-16 years, all self-identifying as Maori, were recruited from their previous participation in a trial of the same intervention. Participants from a previous study were invited if in the mild-to-moderate range on a patient health questionnaire (with scores of 10 to 19 inclusive) and at low risk of self-harm. They then completed a baseline measure CDRS-R [47], producing a | Semistructured interviews analyzed using the inductive approach of thematic analysis [53]. | 5 overall themes were recorded: (1) SPARX was helpful due to its ability to teach-CBT skills for everyday life; (2) Maori involvement in design assisted overall engagement; (3) SPARX characters provided hope and help; (4) SPARX gameplay was enjoyable and challenging; (5) the booklet was useful for thoughts and feelings. |

| | | | | | |
|---|---|---|---|---|---|
| | | | mean score of 49.43 (SD 9.86) | | |
| 7. Tunney et al [55] (Ireland) | *Mindful gNATs* is a free mobile app that mirrors some of the mindfulness-based CBT content within *Pesky gNATs* [56]. However, *Mindful gNATs* can be applied to both clinical and nonclinical populations in any setting. | As part of the design of a cCBT game (*Pesky gNATs*), this study aimed to explore young people's experiences of mindfulness exercises when delivered both in face-to-face settings and through a computer game character. | Overall, 93 young people (56% male) aged 10-12 years (mean 11.07, SD 0.70) were recruited using a convenience sample from 2 Irish primary schools. | A 2-armed qualitative focus groups design was used for participants engaging in mindfulness either via face to face or via a computer game. All data were analyzed using thematic analysis [53]. | 6 overall themes were reported: (1) relaxation; (2) engagement; (3) awareness; (4) thinking; (5) practice; and (6) directing attention. Subthemes from computer-only group were as follows: (1) focus; (2) peaceful; (3) pace; (4) impact on outcome; (5) flowing; (6) metacognition; (7) difficulty; and (8) objects. |
| 8. Chapman et al [57] (the United Kingdom) | *Pesky gNATs* [56]—a 7-level CBT 3D computer game for young people experiencing low mood and/or anxiety. Played in-session by the young person with their therapist. | To record feedback about the benefits and disadvantages of completing cCBT | Young people (female, n=7) aged 13-16 years recruited from a British Tier 3 Child and Adolescent mental health service setting (n=11). | Qualitative thematic analysis [53] was applied to semistructured feedback gathered verbally during the last session. | For benefits of cCBT, participants reported the following: gaining skills of negative thought recognition and relaxation, benefits of using a computer to deliver intervention, game was relaxed and fun, and feeling understood. For areas of improvement of cCBT, participants reported the following: using less information and making |

| | | | | | |
|---|---|---|---|---|---|
| | | | | | the game simpler, increasing age appropriateness, and difficulty of short-term work. |
| 9. Salloum et al [58] (the United States) | *Camp-Cope-A-Lot*—computer-assisted 12-level program focusing on skill-building and exposure-based sessions to address anxiety. During level 3 and 7, therapists meet with parents to offer psychoeducation about exposure approaches to childhood anxiety. | To explore stakeholder perceptions of using cCBT for childhood anxiety in community mental health settings and identify factors that could impact implementation | Six young people (2 female) aged between 7 and 13 years (mean 10.67, SD 2.42) were recruited from community settings. | One focus group and semistructured interviews. Interview guides were developed using previous research on the intervention participants had experienced [59] and were analyzed using thematic analysis and triangulation according to Padgett [60]. | The following themes were found for young people (but also parents and therapists): (1) positive receptiveness; (2) treatment components; (3) therapist factors; (4) applicability of treatment; (5) treatment content. |

| | | | | | |
|---|---|---|---|---|---|
| 10. Lenhard et al [61] (Sweden) | *BiP OCD* delivered via an internet platform containing chapters offering psychoeducative texts, films, and CBT exercises including exposure exercise cognitive interventions. Clinicians can have regular contact with the participant via this platform and can interact by way of comments and feedback. | To describe the experiences of internet-delivered CBT (ICBT) in adolescents with obsessive compulsive disorder (OCD). | Adolescents aged 12-17 years (n=8; 50% male, 50% female) from an open trial on ICBT. Inclusion criteria included a primary diagnosis of OCD, and a total score of 16 or more on the Young people's Yale-Brown Obsessive-Compulsive Scale [62]. | Semistructured interviews as defined by Seidman et al [63], using thematic analysis described by Schilling [64] | 2 overarching themes reported were autonomy and support, with 3 primary subthemes therein: (1) autonomy—self-efficacy, flexibility, secure self-disclosure; (2) support—clinician support, parental support, and identification/normalization. |
| **Tech-assisted CBT for trauma and self-harm** | | | | | |
| 11. Kruger and Swanepoel [65] (South Africa) | Digital art trauma therapy (integrated into a cognitive behavioral meta-model)—10 individual weekly sessions per participant, using a computer | To describe adolescent construction of meaning through digital metaphoric imagery in CBT trauma therapy | Female (court-ordered) residents of a safeguarding unit for traumatized females, aged between 13 and 15 years (n=4) | In-depth case study analysis, including semi-structured interviews, using social constructionism | Participants attached multilayered meaning to their trauma through the use of digital metaphoric imagery. This facilitated post-trauma growth. |

| | | | | | |
|---|---|---|---|---|---|
| | laptop with 2 Art software packages (Windows Paint and ArtRage) | | | | |
| 12. Grist et al [66] (the United Kingdom) | *BlueIce* is a mobile phone app containing a personalized toolbox of strategies to support young people who self-harm (or have self-harmed) between face-to-face CAMHS sessions. Theoretically informed by dialectical behavioral therapy, CBT, and mindfulness, the app includes a mood diary, mood-lifting activities, and automatic safety checks (with options for routing to emergency services | To explore the acceptability, use, and safety of a mobile phone app for young people and adolescent mental health service (CAMHS) users who self-harm (or have self-harmed) | A total of 33 individuals were referred from clinicians due to self-harming concerns. Participants (90% female) were aged between 12 and 17 years (mean 15.98, SD 1.37). | Semi-structured *post-familiarization* interviews were conducted and analyzed using thematic analysis [53]. | 6 key themes emerged from interviews: (1) appraisal of *BlueIce*; (2) usability of *BlueIce*; (3) safety; (4) benefits of *BlueIce*; (5) agency and control; and (6) *BlueIce* less helpful. |

| | | | | | |
|---|---|---|---|---|---|
| | contingent upon self-reported self-harm data). | | | | |
| **Tech-assisted CBT for physical difficulties** | | | | | |
| 13. Nieto et al [67] (Spain) | *DARWeb*—an web-based self-guided, 7-week psychosocial intervention for young people with FAP and their parents | To describe the experiences of an online intervention for young people with functional abdominal pain (FAP) | Young people with FAP aged 9-15 years (n=9; male, n=5) | Semi-structured interviews analyzed using inductive content analysis | 6 main organizing themes were reported: (1) satisfaction; (2) ideas for improvement; (3) burden; (4) pain perception; (5) skills; and (6) general perceptions about online interventions. Participants were generally satisfied with and would recommend the intervention; adding exercises, games, and forums. |

| | | | | | |
|---|---|---|---|---|---|
| 14 Law et al [68] (the United States) | *Web-based Management for Adolescent Pain*—an 8, 30-min series of CBT modules completed over 8-10 weeks, accessed online on desktop or mobile | To describe youth perspectives on successful components of an ICBT intervention for headaches | Young people from randomized controlled trial, aged 11-17 years (n=11; all female) with self-reported chronic idiopathic pain present over the preceding 3 months | Semi-structured interview using inductive thematic analysis [53] | 4 organizing themes were reported: (1) core treatment components; (2) secondary treatment components; (3) general program structure; and (4) suggestions for new components. Subthemes: (1) pain education content was too broad; (2) relaxation training had beneficial strategies; (3) cognitive skills were used and helpful; (4) school intervention was helpful; (5) desire for family-based treatment approach; (6) appreciation for multiple treatment components; and (7) dissatisfaction with the focus of the program. |
