# Supplementary figures and images for: Qualitative Synthesis of Young People’s Experiences With Technology-Assisted Cognitive Behavioral Therapy: Systematic Review
Source: J Med Internet Res. 2019 Nov 12;21(11):e13540. doi: 10.2196/13540 (PMC6880234; doi:10.2196/13540)

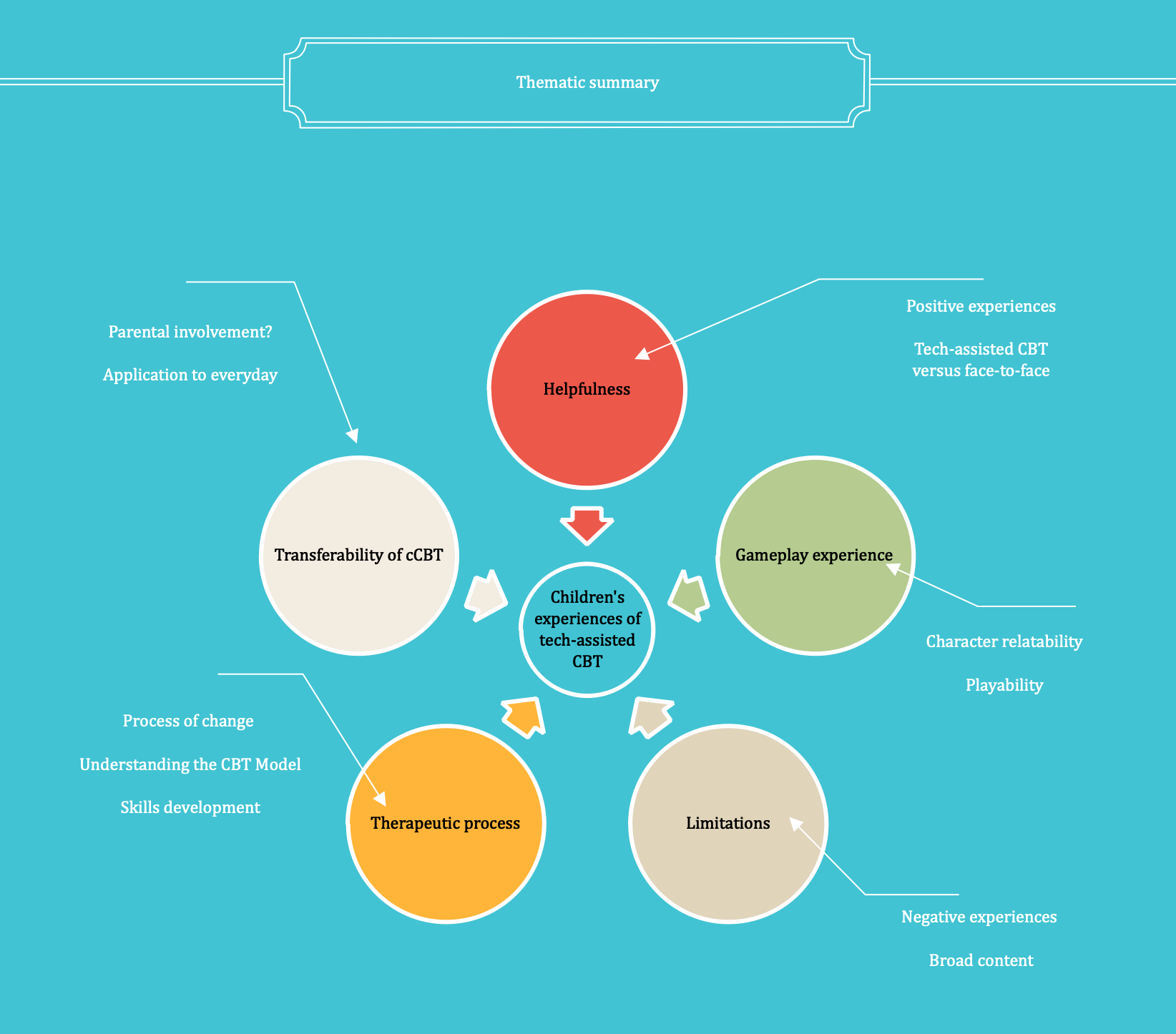

Supplement: Multimedia Appendix 3 [file jmir_v21i11e13540_app3.png]
